# Effects of combined oral contraceptives and metformin on paraoxonase 1 lactonase activity and status in patients with polycystic ovary syndrome and insulin resistance

**DOI:** 10.3389/fendo.2025.1725822

**Published:** 2026-01-13

**Authors:** Jiagui Liang, Qiuyi Wang, Dong Liu, Lukanxuan Wu, Xinyu Qiao, Ruiying Wang, Yuchan Zhong, Wenjie Bo, Huiqiao Lai, Ping Fan, Wei Huang

**Affiliations:** 1 Department of Obstetrics and Gynecology, West China Second University Hospital, Sichuan University, Chengdu, Sichuan, China; 2 Division of Reproductive Medicine, West China Second University Hospital of Sichuan University, Chengdu, Sichuan, China; 3 Laboratory of Genetic Disease and Perinatal Medicine, Key Laboratory of Birth Defects and Related Diseases of Women and Children, Ministry of Education, West China Second University Hospital, Sichuan University, Chengdu, Sichuan, China

**Keywords:** polycystic ovary syndrome, paraoxonase 1, lactonase activity, oral contraceptive, metformin, oxidative stress

## Abstract

**Objective:**

To investigate the effects of combined oral contraceptives (COCs) and metformin treatment on the lactonase activity and status of paraoxonase 1 (PON1), and oxidative stress levels in patients with polycystic ovary syndrome (PCOS) and insulin resistance (IR).

**Design:**

A prospective, self-controlled study.

**Methods:**

Sixty patients diagnosed with PCOS and IR underwent three months of comprehensive therapy, including lifestyle modification, oral metformin (1000–1500 mg/day), and a COC (3 mg drospirenone and 20 µg ethinyl estradiol). Clinical data and blood samples were collected at baseline and after three months of treatment. PON1 levels, lactonase activity, and normalized lactonase activity (NLA), along with *PON1* Q192R and C-108T genetic polymorphisms, oxidative stress markers, hormonal profiles, and metabolic parameters, were analyzed.

**Results:**

After treatment, significant decreases were observed in body mass index (BMI), Global Acne Grading System scores, androstenedione, fasting insulin, the homeostasis model assessment of insulin resistance index, and total antioxidant capacity (*P* < 0.05). In contrast, serum PON1 lactonase activity, apolipoprotein (apo)A1, triglycerides, and 2-h glucose levels were significantly increased (*P* < 0.05). Spearman’s correlation analysis showed that lactonase activity and PON1 status were correlated with serum apoA1, high-density lipoprotein cholesterol (HDL-C), low-density lipoprotein cholesterol, total oxidant status, and the oxidative stress index (*P* < 0.05). Moreover, the group with improved NLA showed higher PON1 lactonase activity and HDL-C levels, whereas the group without improved NLA showed higher PON1 levels after treatment.

**Conclusion:**

Treatment with COCs and metformin enhanced the antioxidant capacity of circulating HDL and improved BMI, glycolipid metabolism, IR, and hyperandrogenism in patients with PCOS and IR.

**Clinical Trial Registration:**

https://www.chictr.org.cn/showproj.html?proj=152682, identifier ChiCTR2200057114.

## 1 Introduction

Polycystic ovary syndrome (PCOS) is a highly heterogeneous reproductive, endocrine, and metabolic disorder (1, 2), which affects 5-18% of women of reproductive age (3). Although its exact pathogenesis remains elusive, its development and progression are closely associated with oxidative stress, overweight/obesity, insulin resistance (IR), and metabolic abnormalities (1, 4–7).

Paraoxonase 1 (PON1) is an antioxidant enzyme that functions primarily in association with high-density lipoproteins (HDL) (8, 9). It exhibits multiple enzymatic activities, including paraoxonase, arylesterase, lactonase, and homocysteine thiolactonase (8, 10). It can hydrolyze various exogenous and endogenous substrates, such as organophosphates, aryl esters, lactones, and lipid peroxides (8). In addition, it can degrade many oxidative stress products (8, 11). Owing to these activities, PON1 exhibits antioxidant effects. It is closely associated with various diseases and biological processes, including atherosclerosis, cardiovascular diseases, diabetes, neurodegenerative disorders, conditions related to oxidative stress and inflammation, infections, and cancers (12, 13). Its beneficial role in the pathophysiology of these disorders, along with its potential mechanisms of action, has been extensively studied and reported (12–15).

Studies have demonstrated that the antioxidative and anti-inflammatory properties of PON1 result from its lactonase activity, which enables it to hydrolyze lipid peroxides and active derivatives of arachidonic acid, such as eicosanoids (8, 13). In addition, the ratio of PON1 lactonase activity to PON1 level, known as normalized lactonase activity (NLA), may reflect the catalytic stimulation of PON1 lactonase by HDL (9, 15). Therefore, measuring both lactonase activity and NLA provides a better assessment of PON1’s antioxidant, anti-inflammatory, and anti-atherogenic functions (13–15).

Oxidative stress and metabolic dysfunction play crucial roles in the pathogenesis of PCOS (1, 4, 5). Patients with PCOS exhibit increased oxidative stress and impaired antioxidant and anti-inflammatory function of HDL (5, 6, 16). Studies have reported increased PON1 lactonase activity alongside decreased PON1 paraoxonase activity in patients with PCOS (15, 17, 18). Genetic variants of the *PON1* gene, such as Q192R (rs662) and C-108T (rs705379) polymorphisms, have a considerable impact on the activities and enzyme levels of PON1 (14, 15, 19). Furthermore, PON1 paraoxonase and arylesterase activities have been shown to increase in patients with PCOS following treatment with metformin and/or COCs (20, 21). However, the effects of such treatments on PON1 lactonase activity and NLA in PCOS remain unclear. In this study, we investigated changes in PON1 lactonase activity and overall PON1 status, along with metabolic, oxidative stress, and clinical indicators before and after combined treatment, including lifestyle modification, COCs, and metformin in 60 patients with PCOS and IR. We also analyzed the *PON1* Q192R and C-108T genetic polymorphism in the study population.

## 2 Materials and methods

### 2.1 Study population

This prospective self-comparison study was approved by the Institutional Review Board of the West China Second University Hospital, Sichuan University (Wei Huang: 2021-113), and was performed in line with the principles of the Declaration of Helsinki. The participants with diagnosed with PCOS were recruited between January 2021 and March 2025. Informed consent was obtained from all participants. The diagnosis of PCOS was based on the Rotterdam Criteria 2003 (2). IR was defined as a homeostasis model assessment of insulin resistance index (HOMA−IR) > 2.69 (22, 23). Participants were excluded from the study if they met any of the following criteria: i) use of sex hormone-related or metabolic drugs within the past three months; ii) presence of uncontrolled diabetes, thyroid diseases, or similar conditions; iii) systemic diseases such as cardiovascular disorders, liver and kidney dysfunction, immune system disorders, malignancies; and iv) smoking or alcohol consumption. Finally, 60 patients with PCOS and IR were included in this study. The mean age and body mass index (BMI) of the study participants were 25.88 ± 2.82 (19.00–34.00) years and 26.24 ± 4.48 (18.86–39.31) kg/m^2^, respectively.

Demographic and anthropometrics information of all participants, including age, menstrual status, childbearing history, family history, height, weight, waist circumference, hip circumference, systolic blood pressure (SBP), diastolic blood pressure (DBP), BMI, and waist-to-height ratio, were measured or estimated. Hyperandrogenic symptoms were evaluated based on the Modified Ferriman-Gallwey (F-G) score of hirsutium and the Global Acne Grading System (GAGS) of acne (24–26).

During the first visit, peripheral blood samples were collected from each participant after an overnight fast. A 75g oral glucose tolerance test (OGTT) was performed immediately after fasting blood samples were collected. Plasma and serum samples were used for hormonal and metabolic analyses, and additional aliquots were stored at −80°C for future investigations.

Serum estradiol (E_2_), progesterone (P), testosterone (T), luteinizing hormone (LH), follicle-stimulating hormone (FSH), and fasting insulin (Ins) levels were measured using chemiluminescence immunoassays (ADVIA Centaur; Siemens). Fasting glucose (Glu) levels were measured using a hexokinase assay (ADVI2400; Siemens). The HOMA−IR was calculated as described previously (7). Serum total cholesterol (TC), triglyceride (TG), low-density lipoprotein cholesterol (LDL-C), and HDL cholesterol (HDL-C) levels were measured using enzymatic assays (ADVIA 2400; Siemens).

All participants were advised to follow a healthy lifestyle, which included a balanced diet and at least 150 min of aerobic exercise per week. In addition, they were prescribed a daily COC containing 3 mg drospirenone and 20 µg ethinyl estradiol. Metformin treatment was initiated at a low dose of 500 mg/day and gradually increased to 1000–1500 mg/day for participants with IR. The lifestyle modification and medication administration regimen were maintained continuously for three months. Afterward, participants returned for follow-up assessments, which included evaluation of menstrual cycles, hyperandrogenic symptoms and score, anthropometric measurements, OGTT, and endocrine and metabolic parameters.

### 2.2 Analysis of oxidative stress markers, PON1 activities, and *PON1* Q192R and C-108T genetic polymorphisms

The serum total oxidant status (TOS) was measured as previously described (6). Total antioxidant capacity (T-AOC) and malondialdehyde (MDA) levels were determined by spectrophotometry using T-AOC and micro-MDA kits (Nanjing Jiancheng Bioengineering Institute, Nanjing, China), respectively. The oxidative stress index (OSI) is expressed as the ratio of TOS to T-AOC (6).

The lactonase activity, level, and NLA of PON1 were assessed using the methods and procedures described in our previous study (15). Serum PON1 lactonase activity was measured using an automated microplate enzyme kinetic colorimetric assay, with 5-thiobutyl butyrolactone (TBBL) as the substrate. The reaction was monitored by measuring the optical density of thiol-containing products at 412 nm in the presence of 5’5 dithio-bis 2-nitrobenzoic acid (DTNB) over 5 min at 25°C (ε = 7000 OD/M). The lactonase activity was expressed in units per milliliter (U/ml), where one unit corresponds to the hydrolysis of TBBL per min per milliliter of undiluted serum. Serum PON1 levels were determined by measuring the hydrolysis of the substrate 7-O-diethylphosphoryl-3-cyano-4-methyl-7-hydroxycoumarin (DEPCyMC). The reaction product, 3-Cyano-4-methyl-7-hydroxycoumarin, was quantified by absorbance at 400 nm over 5 min at 25°C using an automated microplate reader (ε = 22240 OD/M). Enzyme activities (levels) are expressed as mU/mL (1 mU = 1 nmol of DEPCyMC hydrolyzed per min per ml of undiluted serum). The NLA of PON1 was calculated using the following equation.

NLA = TBBL activity (U/mL) × 1000 / DEPCyMC activity (mU/mL).

The intra- and inter-assay coefficients of variation for all the tests were < 5% and 10%, respectively.

The genomic DNA samples were extracted from peripheral blood of the participants. The *PON1* Q192R and C-108T polymorphisms were genotyped as previously described (27, 28).

### 2.3 Statistical analyses

Data were analyzed using SPSS 26.0. A paired-sample t-test was used to evaluate differences in the variables. Spearman correlation analysis was used to determine the correlations between PON1 level, lactonase activity, and NLA and various clinical, metabolic, and oxidative stress indicators. The chi-square test was employed to examine differences in genotype distributions. A value of *P* < 0.05 was considered statistically significant.

## 3 Results

### 3.1 Clinical and biochemical characteristics of PCOS women before and after treatment

A total of 60 patients with PCOS completed the 3-month treatment and follow-up period. Before treatment, the mean serum hormone levels were as follows: E_2_, 269.68 ± 175.59 pmol/L; P, 4.49 ± 7.07 nmol/L; T, 1.22 ± 0.37 nmol/L; LH, 12.47 ± 9.68 IU/L; FSH, 5.95 ± 1.80 IU/L; and the LH/FSH ratio, 2.03 ± 1.23.

The baseline clinical and biochemical parameters of the participants are presented in **Table 1**. After three months of treatment, several significant improvements were observed: BMI decreased from 26.24 ± 4.48 kg/m^2^ to 25.08 ± 3.96 kg/m^2^, mean menstrual cycle length shortened from 53.85 ± 24.61 days to 30.70 ± 9.82 days, GAGS score declined from 2.77 ± 3.57 to 1.69 ± 2.37, and androstenedione levels decreased from 14.15 ± 6.19 nmol/L to 11.58 ± 4.89 nmol/L, as well as fasting Ins and HOMA-IR, which showed significant reductions (*P* < 0.01), and apoA1 levels exhibited a significant increase (*P* < 0.001). Conversely, levels of TG, and 2-h Glu were significantly elevated (*P* < 0.001). Meanwhile, no significant differences were observed in the Ferriman-Gallwey score and dehydroepiandrosterone sulfate levels after treatment (*P* > 0.05). Our study indicates that patients with PCOS have improved overweight/obesity, menstrual cycle, hyperandrogenic status, and some glycolipid metabolism parameters after 3 months of combined treatment with lifestyle, COCs, and metformin.

**Table 1:** Clinical and biochemical characteristics of the participants.

	Baseline	After treatment	*P*
Clinical characteristics			
BMI (kg/m^2^)	26.24 ± 4.48	25.08 ± 3.96	< 0.001
Waist-to-height ratio	0.50 ± 0.07	0.51 ± 0.09	0.457
SBP (mmHg)	112.23 ± 11.39	110.53 ± 10.22	0.248
DBP (mmHg)	75.12 ± 8.54	74.75 ± 9.26	0.750
Mean menstrual cycle (days)	53.85 ± 24.61	30.70 ± 9.82	< 0.001
Ferriman-Gallwey score	2.45 ± 2.07	2.40 ± 1.68	0.775
GAGS	2.77 ± 3.57	1.69 ± 2.37	0.001
Metabolic profile			
Fasting Glu (mmol/L)	5.16 ± 0.60	5.01 ± 0.69	0.105
Fasting Ins (pmol/L)	136.69 ± 70.41	114.31 ± 56.91	0.002
HOMA-IR	4.63 ± 2.74	3.52 ± 2.17	< 0.001
2-h Glu (mmol/L)	7.74 ± 2.20	8.64 ± 2.15	< 0.001
2-h Ins (pmol/L)	1159.47 ± 658.51	1112.12 ± 559.21	0.422
TC (mmol/L)	4.51 ± 0.84	4.66 ± 0.82	0.115
TG (mmol/L)	1.49 ± 0.76	1.99 ± 1.11	< 0.001
HDL-C (mmol/L)	1.23 ± 0.31	1.28 ± 0.30	0.062
LDL-C (mmol/L)	3.06 ± 0.82	3.04 ± 0.91	0.832
ApoA1 (g/L)	1.36 ± 0.18	1.50 ± 0.25	< 0.001
ApoB (g/L)	0.84 ± 0.21	0.84 ± 0.21	0.686
DHEA-S (µmol/L)	7.31 ± 2.96	7.81 ± 2.69	0.157
AND (nmol/L)	14.15 ± 6.19	11.58 ± 4.89	0.001

Values are presented as the mean ± SD.

AND, androstenedione; Apo, apolipoprotein; BMI, body mass index; DBP, diastolic blood pressure; DHEA-S, dehydroepiandrosterone sulfate; GAGS, Global Acne Grading System; Glu, glucose; HDL-C, high-density lipoprotein cholesterol; HOMA-IR, homeostasis model insulin resistance index; Ins, insulin; LDL-C, low-density lipoprotein cholesterol; SBP, systolic blood pressure; TC, total cholesterol; TG, triglyceride.

### 3.2 PON1 activities and oxidative stress indicators

As shown in **Table 2**, PON1 lactonase activity and OSI increased significantly (*P* < 0.01), the NLA of PON1 tended to increase (*P* = 0.072), and the T-AOC levels significantly decreased (*P* = 0.005) after treatment. The changes in TOS, MDA, and PON1 levels before and after treatment were not statistically significant (*P* > 0.05). In short, the PON1 lactonase activity and the relative oxidative stress level (OSI) were significantly increased in these patients after treatment.

**Table 2:** Oxidative stress and PON1 indicators of the participants.

	Baseline	After treatment	*P*
TOS (µmol H_2_O_2_ Equiv./L)	15.97 ± 7.19	16.25 ± 4.88	0.807
T-AOC (U/ml/min)	18.05 ± 5.08	15.54 ± 5.76	0.005
OSI	0.92 ± 0.39	1.21 ± 0.69	0.009
MDA (nmol/ml)	4.14 ± 0.86	4.13 ± 0.64	0.931
PON1 lactonase activity (U/ml)	14.72 ± 3.75	15.83 ± 5.18	0.006
PON1 level (mU/ml)	40.94 ± 7.46	41.71 ± 8.52	0.383
NLA	365.50 ± 91.49	385.27 ± 108.22	0.072

Values are presented as the mean ± SD.

MDA, malondialdehyde; NLA, normalized lactonase activity; OSI, oxidative stress index; PON1, paraoxonase 1; T-AOC, total antioxidant capacity; TOS, total oxidant status.

### 3.3 Correlation between PON1 activities and clinical and biochemical parameters

We analyzed the correlation between the levels and activities of PON1 and the clinical and biochemical parameters before and after treatment using Spearman correlation analysis.

**Table 3** shows that PON1 lactonase activity was positively correlated with HDL-C and apoA1 levels both before and after treatment (*P* < 0.05). PON1 levels were negatively correlated with TOS before treatment (*P* = 0.028), whereas PON1 NLA was positively correlated with HDL-C levels before and after treatment (*P* < 0.05) and positively correlated with TOS and OSI before treatment (*P* < 0.05). Our study shows that PON1 lactonase activity and status are mainly related to HDL-C, apoA1, and oxidative stress levels in the study population.

**Table 3:** Correlations between PON1 activities and other indicators before and after treatment.

	PON1 lactonase activity (U/ml)		PON1 level (mU/ml)		NLA	
	Baseline	After treatment	Baseline	After treatment	Baseline	After treatment
BMI (kg/m^2^)	-0.064	-0.089	-0.054	-0.012	0.024	0.012
Waist-to-height ratio	0.048	0.050	0.015	-0.050	0.070	0.123
SBP (mmHg)	0.157	0.013	0.127	0.046	0.048	-0.021
DBP (mmHg)	-0.003	0.088	0.017	0.119	-0.002	0.061
Mean menstrual cycle (days)	0.084	0.050	0.077	-0.034	-0.010	0.035
Ferriman–Gallwey score	0.210	0.065	-0.026	-0.143	0.226	0.195
GAGS	0.209	0.111	0.251	0.203	-0.043	-0.028
DHEA-S (µmol/L)	-0.080	0.121	0.092	0.179	-0.179	-0.024
AND (nmol/L)	-0.016	-0.045	-0.179	-0.105	0.052	-0.030
Fasting Glu (mmol/L)	0.042	-0.121	-0.054	-0.196	0.034	0.043
Fasting Ins (pmol/L)	0.183	-0.016	-0.032	0.146	0.077	-0.084
HOMA-IR	0.172	-0.025	-0.030	0.037	0.061	-0.002
2-h Glu (mmol/L)	0.132	-0.010	-0.014	0.016	0.106	0.031
2-h Ins (pmol/L)	0.194	0.107	-0.045	0.118	0.082	-0.015
TC (mmol/L)	0.202	0.004	0.019	0.082	0.187	-0.033
TG (mmol/L)	-0.092	-0.062	-0.156	0.024	0.049	-0.065
HDL-C (mmol/L)	0.368**	0.381**	0.087	0.101	0.274*	0.276*
LDL (mmol/L)	-0.018	-0.268*	-0.056	-0.104	-0.003	-0.180
ApoA1 (g/L)	0.307*	0.347**	0.023	0.220	0.207	0.158
ApoB (g/L)	-0.071	-0.139	-0.175	-0.191	0.030	0.026
TOS (µmol H_2_O_2_ Equiv./L)	-0.009	0.167	-0.284*	0.149	0.272*	0.049
OSI	0.004	0.201	-0.222	0.099	0.276*	0.072
T-AOC (U/ml/min)	-0.027	-0.145	-0.014	0.016	-0.027	-0.073
MDA (nmol/ml)	0.154	0.043	-0.049	0.102	0.215	-0.090

AND, androstenedione; Apo, apolipoprotein; BMI, body mass index; DBP, diastolic blood pressure; DHEA-S, dehydroepiandrosterone sulfate; GAGS, Global Acne Grading System; Glu, glucose; HDL-C, high-density lipoprotein cholesterol; HOMA-IR, homeostasis model insulin resistance index; Ins, insulin; LDL-C, low-density lipoprotein cholesterol; MDA, malondialdehyde; NLA, normalized lactonase activity; OSI, oxidative stress index; PON1, paraoxonase 1; SBP, systolic blood pressure; T-AOC, total antioxidant capacity; TC, total cholesterol; TG, triglyceride; TOS, total oxidant status.

*P < 0.05;**P < 0.01

### 3.4 Effects of PON1 NLA changes on clinical and biochemical parameters

Based on whether PON1 NLA increased after treatment, participants were divided into two groups: those with improved NLA and those without. The impact of changes in PON1 NLA on clinical and biochemical indicators was then further analyzed (**Table 4**).

**Table 4:** Clinical and biochemical characteristics of the groups with improved NLA or not.

	The group with improved NLA (n = 42)				The group without improved NLA (n = 18)			
	Baseline	After treatment	*P*	Cohen's ***d*** [95%CI]	Baseline	After treatment	*P*	Cohen's ***d*** *[95%CI]
Clinical characteristics								
BMI (kg/m^2^)	25.67 ± 4.42	24.61 ± 3.81	0.005	2.33 [0.13, 0.77]	27.56 ± 4.45	26.17 ± 4.19	<0.001	1.28 [0.49, 1.65]
Waist-to-height ratio	0.50 ± 0.06	0.51 ± 0.09	0.549	0.08 [-0.40, 0.21]	0.52 ± 0.09	0.52 ± 0.09	0.631	0.05 [-0.56, 0.34]
SBP (mmHg)	112.95 ± 11.19	110.05 ± 10.48	0.098	11.11 [-0.05, 0.57]	110.56 ± 12.02	111.67 ± 9.80	0.687	11.75 [-0.55, 0.36]
DBP (mmHg)	74.57 ± 7.67	73.62 ± 7.01	0.404	7.32 [-0.17, 0.43]	76.39 ± 10.43	77.39 ± 12.98	0.725	12.15 [-0.53, 0.37]
Ferriman–Gallwey score	2.50 ± 2.18	2.36 ± 1.66	0.551	1.54 [-0.21, 0.40]	2.33 ± 1.85	2.50 ± 1.76	0.331	0.72 [-0.69, 0.23]
GAGS	3.31 ± 3.89	1.94 ± 2.52	0.003	2.81 [0.16, 0.80]	1.50 ± 2.31	1.11 ± 1.90	0.144	1.10 [-0.12, 0.81]
Biochemical parameters								
Fasting Glu (mmol/L)	5.02 ± 0.51	4.96 ± 0.78	0.580	0.71 [-0.22, 0.39]	5.49 ± 0.68	5.13 ± 0.44	0.041	0.71 [0.02, 0.98]
Fasting Ins (pmol/L)	127.21 ± 64.98	106.94 ± 53.84	0.012	49.87 [0.09, 0.72]	158.81 ± 79.24	131.49 ± 61.68	0.071	61.55 [-0.04, 0.91]
HOMA-IR	4.13 ± 2.17	3.15 ± 2.03	0.008	2.26 [0.11, 0.75]	5.82 ± 3.54	4.38 ± 2.30	0.030	2.63 [0.05, 1.03]
2-h Glu (mmol/L)	7.40 ± 1.67	8.76 ± 2.10	<0.001	1.73 [-1.13, -0.44]	8.53 ± 2.54	8.37 ± 2.32	0.756	2.15 [-0.38, 0.52]
2-h Ins (pmol//L)	1100.78 ± 678.84	1092.58 ± 557.97	0.899	414.14 [-0.28, 0.32]	1296.39 ± 604.32	1157.73 ± 575.59	0.288	548.41 [-0.21, 0.71]
TC (mmol/L)	4.45 ± 0.82	4.59 ± 0.78	0.257	0.79 [-0.48, 0.13]	4.64 ± 0.89	4.82 ± 0.90	0.225	0.61 [-0.75, 0.18]
TG (mmol/L)	1.48 ± 0.86	1.92 ± 1.17	0.007	1.02 [-0.75, -0.12]	1.52 ± 0.49	2.15 ± 0.98	0.006	0.88 [-1.22, -0.20]
HDL-C (mmol/L)	1.20 ± 0.29	1.28 ± 0.33	0.020	0.21 [-0.69, -0.06]	1.29 ± 0.35	1.28 ± 0.25	0.898	0.22 [-0.42, 0.48]
LDL-C (mmol/L)	3.00 ± 0.74	2.99 ± 0.84	0.907	0.78 [-0.28, 0.32]	3.18 ± 1.00	3.14 ± 1.06	0.813	0.60 [-0.40, 0.51]
ApoA1 (g/L)	1.35 ± 0.15	1.47 ± 0.25	<0.001	0.22 [-0.90, -0.25]	1.40 ± 0.23	1.56 ± 0.26	0.003	0.20 [-1.31, -0.26]
ApoB (g/L)	0.82 ± 0.20	0.82 ± 0.21	0.924	0.14 [-0.29, 0.32]	0.86 ± 0.21	0.90 ± 0.20	0.461	0.19 [-0.63, 0.28]
DHEA-S (µmol/L)	7.61 ± 3.04	7.98 ± 2.70	0.382	2.74 [-0.44, 0.17]	6.61 ± 2.70	7.41 ± 2.71	0.222	2.72 [-0.75, 0.17]
AND (nmol/L)	14.05 ± 6.61	11.74 ± 4.56	0.016	5.97 [0.07, 0.70]	14.39 ± 5.27	11.22 ± 5.69	0.039	6.15 [0.03, 0.99]
TOS (µmol H_2_O_2_ Equiv./L)	15.68 ± 6.34	15.79 ± 4.74	0.930	8.44 [-0.32, 0.29]	16.64 ± 9.03	17.32 ± 5.15	0.784	10.46 [-0.52, 0.39]
OSI	0.92 ± 0.40	1.24 ± 0.78	0.031	0.93 [-0.65, -0.03]	0.91 ± 0.37	1.13 ± 0.40	0.115	0.57 [-0.85, 0.09]
T-AOC (U/ml/min)	18.05 ± 5.50	15.31 ± 6.26	0.025	7.67 [0.04, 0.67]	18.04 ± 4.09	16.09 ± 4.48	0.024	3.41 [0.08, 1.06]
MDA (nmol/ml)	4.06 ± 0.75	4.08 ± 0.55	0.870	0.88 [-0.33, 0.28]	4.34 ± 1.06	4.24 ± 0.83	0.783	1.42 [-0.39, 0.52]
PON1 lactonase activity (U/ml)	14.53 ± 3.04	16.18 ± 3.99	<0.001	2.97 [-0.88, -0.23]	15.18 ± 5.13	15.01 ± 7.32	0.800	2.79 [-0.39, 0.51]
PON1 level (mU/ml)	42.45 ± 7.50	41.42 ± 8.26	0.260	5.81 [-0.13, 0.48]	37.41 ± 6.22	42.37 ± 9.32	0.010	7.45 [-1.16, -0.16]

Values are presented as the mean ± SD. Paired samples t-test was performed for statistical analysis.

*The Hedges’ correction is commonly employed to evaluate situations where the sample size is less than 30.

AND, androstenedione; Apo, apolipoprotein; BMI, body mass index; CI, confidence interval; DBP, diastolic blood pressure; DHEA-S, dehydroepiandrosterone sulfate; GAGS, global acne grading system; Glu, glucose; HDL-C, high-density lipoprotein cholesterol; HOMA-IR, homeostasis model insulin resistance index; Ins, insulin; LDL-C, low-density lipoprotein cholesterol; MDA, malondialdehyde; NLA, normalized lactonase activity; OSI, oxidative stress index; PON1, paraoxonase 1; SBP, systolic blood pressure; T-AOC, total antioxidant capacity; TC, total cholesterol; TG, triglyceride; TOS, total oxidant status

The results showed that both groups experienced significant reductions in BMI, androstenedione, and T-AOC (*P* < 0.05), as well as significant increases in apoA1 and TG levels (*P* < 0.01) after treatment. In the group with improved NLA, HDL-C, PON1 lactonase activity, 2-h Glu, and OSI significantly increased (*P* < 0.05), whereas GAGS, fasting Ins levels, and HOMA-IR significantly decreased (*P* < 0.05) after treatment. In the group without improved NLA, fasting Glu levels and HOMA-IR were decreased (*P* < 0.05), while PON1 levels were elevated (*P* = 0.010); however, HDL-C and PON1 lactonase activity were not significantly different (*P* > 0.05) after treatment. In brief, the increase in PON1 NLA is associated with the elevation of PON1 lactonase activity, accompanied by an increase in both HDL-C and apoA1 levels. However, this phenomenon was not observed in the group without improved NLA, where a significant rise in PON1 levels was noted instead.

Additionally, no significant differences were observed in either genotypes or alleles of *PON1* Q192R and C-108T polymorphisms between the group with improved NLA and the group without improved NLA (*P* > 0.05; **Supplementary Table 1**).

## 4 Discussion

This is the first study to evaluate the effects of lifestyle modification, COCs, and metformin treatment on the lactonase activity and status of PON1 in patients with PCOS and IR. We found that serum lactonase activity and NLA of PON1, TG, 2-h Glu, OSI, and apoA1 levels increased, whereas BMI, fasting Ins concentration, HOMA-IR, and T-AOC decreased, and GAGS and androstenedione levels improved after three months of comprehensive treatment. Moreover, we found that PON1 lactonase activity and status were associated with serum apoA1, HDL-C, LDL-C, TOS, and OSI. Subgroup analysis showed that the group with improved NLA had higher PON1 lactonase activity and HDL-C levels after treatment, whereas the group without improved NLA had higher total PON1 levels. These findings suggest that lifestyle modification, COCs, and metformin treatment not only improves BMI and glycolipid metabolism but also enhances the antioxidant capacity of circulating HDL in patients with PCOS and IR.

PON1 exerts antioxidant and anti-inflammatory effects by hydrolyzing lipid peroxides through its lactonase activity (8, 9, 12). PON1 is secreted by the liver and requires apoA1 and apoE in HDL for complete activation (29, 30). HDL maturation optimizes PON1 (31, 32). HDL particles carrying apoA1 and apoE bind to PON1 with high affinity, stabilizing the enzyme over 100-fold and enhancing its lipolactonase activity (30, 33). The activity of PON1 depends on the optimal association between discrete HDL subclasses and apolipoproteins (31). A Mendelian randomization analysis highlighted low HDL-C as one of the most prominent causal factors for PCOS (34). PON1 not only inhibits LDL oxidation, but also promotes macrophage-associated cholesterol efflux (13, 35). This may explain why, in this study, an increase in PON1 NLA after treatment was accompanied by significant increases in lactonase activity, HDL-C, and apoA1 levels in patients with PCOS. The activity of PON1 measured by DEPCyMC hydrolysis has been shown to be a reliable marker of total PON1 protein levels, as it is unaffected by the state of HDL, or the degree of catalytic stimulation and stability resulting from PON1’s tight binding to HDL-apoA1 (9). In addition, excessive levels of oxidant molecules and hyperglycemia may lead to oxidative damage and glycosylation of PON1, thereby reducing the enzyme activity (36, 37). The -108C/T genetic polymorphism in the promoter region of *PON1* gene is the most important regulatory region variant that affects *PON1* gene expression, and has a significant impact on the level and lactonase activity of PON1 (13, 15). The *PON1* Q/R polymorphism at position 192 affects *PON1* binding to HDL, PON1 lactonase activity, and PON1 NLA (15, 38). In this study, we found that, different from the group with improved NLA, an increase in PON1 enzyme levels and apoA1, but not HDL-C, was not accompanied by an increase in PON1 lactonase activity in the group without improved NLA after treatment. However, we did not observe significant differences in the genotypic and allelic frequencies of *PON1* C-108T and Q192R variants between the groups with and without improved NLA, suggesting that these two polymorphisms may not be the primary drivers of the observed phenomenon. The interaction between HDL maturation, oxidative stress status, and genetic polymorphisms may be an important reason in this study, which deserves further validation in larger cohort studies. In brief, these findings suggest that simultaneous measurement of HDL-C, apoA1, and PON1 lactonase activity provides a more accurate assessment of PON1’s antioxidant function and its response to treatment.

BMI is identified as one of the most prominent causal factors for PCOS and plays a key role in the development of PCOS (4, 34). In this study, BMI significantly decreased after treatment regardless of the grouping criteria used. Additionally, GAGS, and androstenedione levels all showed meaningful clinical reductions, and menstrual regularity significantly improved. Furthermore, BMI was positively correlated with T-AOC (r = 0.419, *P* < 0.001) before treatment, as well as TOS (r = 0.350, *P* < 0.01) after treatment. However, no correlation was found between BMI and the PON1 levels, lactonase activity, or NLA. Consistent with our findings, a previous study reported no significant differences in PON1 activity or its distribution across HDL subclasses between normal-weight and overweight or obese patients with PCOS (39). Weight loss has been shown to reduce oxidative stress and enhance antioxidant capacity (40, 41). In the present study, PON1 NLA was positively correlated with TOS and OSI, while PON1 levels were negatively correlated with TOS before treatment, but not after. These findings support the idea that the elevated PON1 lactonase activities and levels may serve as a compensatory response to increased oxidative stress. Additionally, obesity in PCOS may influence PON1 levels and activity indirectly, through its impact on oxidative stress. Therefore, weight management is crucial in the treatment of patients with PCOS.

Our study also found that after three months of COCs and metformin treatment, serum TG and 2-h Glu levels significantly increased in patients with PCOS, especially in patients with improved PON1 NLA. It has been reported that the use of COC is associated with increased TG levels, but not 2-h Glu levels (42, 43). Although the fasting insulin and HOMA-IR decrease in the patients with improved NLA after treatment in this study, the reason for the increase in 2-h Glu levels remains unclear. Further research is needed to better understand this phenomenon and clarify the underlying mechanisms.

Previous studies have demonstrated that adequate physical activity can enhance insulin sensitivity, improve IR, and reduce androgen levels in patients with PCOS (44). Diet changes in lifestyle are also beneficial for improving inflammation and oxidative stress (45). COCs not only improve sex hormone levels but also exert beneficial effects on HDL-C levels (43). The combination of COCs and metformin is more effective than monotherapy (46). Our study conforms to the 2023 Updated International Evidence-Based Guidelines, which clearly state that for overweight or obese patients with PCOS, the combined use of lifestyle interventions, metformin, and COCs yields favorable short-term outcomes and long-term health benefits (47).

This study has some limitations. First, the sample size was relatively small, especially in subgroup analysis, which might have affected the power of the related parameters or caused a failure to reach statistical significance. Second, the treatment duration in our study may have been relatively short, which could explain why some indicators showed trends toward improvement without reaching statistical significance. Third, we were unable to provide the average number of follicles of the study participants, due to the lack of accurate follicle count in some patients in this study. These limitations warrant further investigation.

In conclusion, treatment with a combination of lifestyle modification, COCs, and metformin not only improves clinical features such as menstrual regularity, hyperandrogenism, BMI, IR, and glycolipid metabolism, but also enhances the antioxidant capacity of circulating HDL by increasing PON1 lactonase activity in patients with PCOS and IR. Furthermore, we found that the compensatory stimulation of PON1 lactonase activity may be partly related to increased oxidative stress in these patients. Our findings also highlight the importance of monitoring both PON1 lactonase activity and HDL-C/apoA1 status in PCOS management. Future studies should explore targeted interventions at enhancing PON1-HDL binding, which may improve antioxidant efficacy in patients who do not respond to conventional therapy.

## Data Availability

The original contributions presented in the study are included in the article/**Supplementary Material**. Further inquiries can be directed to the corresponding authors.
