# Supplementary material for: Effects of combined oral contraceptives and metformin on paraoxonase 1 lactonase activity and status in patients with polycystic ovary syndrome and insulin resistance
Source: Front Endocrinol (Lausanne). 2026 Jan 13;16:1725822. doi: 10.3389/fendo.2025.1725822 (PMC12834811; doi:10.3389/fendo.2025.1725822)
Supplement: Supplementary file 1 [file SupplementaryFile1.docx]

**Supplementary Table 1. Genotypic and allelic frequencies of *PON1* C-108T and Q192R polymorphisms in the groups with improved NLA or not**

|  |  | The group with improved NLA (n = 42) | The group without improved NLA (n = 18) | χ^2^ | | *P* |
| --- | --- | --- | --- | --- | --- | --- |
| C-108T | | | | | | |
| genotype | CC | 15 (35.71%) | 6 (33.33%) |  | |  |
|  | CT | 19 (45.24%) | 6 (33.33%) |  | |  |
|  | TT | 8 (19.05%) | 6 (33.33%) | 1.572 | | 0.480 |
| *P*_HWE_ |  | 0.904 | 0.367 |  | |  |
| allele | C | 49 (58.33%) | 18 (50%) |  | |  |
|  | T | 35 (41.67%) | 18 (50%) | 0.710 | | 0.400 |
| Q192R | | | | | | |
| genotype | QQ | 7 (16.67%) | 3 (16.67%) |  | |  |
|  | QR | 17 (40.48%) | 3 (16.67%) |  | |  |
|  | RR | 18 (42.88%) | 12 (66.67%) | 3.558* | | 0.155 |
| *P*_HWE_ |  | 0.698 | 0.062 |  | |  |
| allele | Q | 31 (35.90%) | 9 (25.00%) |  | |  |
|  | R | 53 (63.10%) | 27 (75.00%) | | 1.607 | 0.205 |

Values are presented as number (%) of the participants with improved NLA or not.

*P*_HWE_: *P* value of Hardy-Weinberg equilibrium test.

* Since 2 cells have expected count less than 5, the Fisher’s exact test was used.
